# ATRA COMPETENCY STUDY: PROMOTING DEVELOPMENT OF RECREATIONAL THERAPISTS SERVING OLDER ADULTS

**DOI:** 10.1093/geroni/igz038.006

**Published:** 2019-11-08

**Authors:** Martha E Kemeny, Brent Hawkins, Heather Porter

**Affiliations:** 1 Slippery Rock University, Slippery Rock, Pennsylvania, United States; 2 Clemson University, Clemson, South Carolina, United States; 3 Temple University, Philadelphia, Pennsylvania, United States

## Abstract

Competency concerns the knowledge, skills, and abilities needed to perform specific job tasks. In the field of recreational therapy (RT), four documents from the certification (NCTRC), accreditation (CARTE), and professional association (ATRA) broadly identify areas of competency for RTs. While broad competencies are defined, no specific competencies (i.e., diagnoses, assessments, interventions, theories) are identified. Sixty-seven RT experts, including 10 verified older adult experts with greater than five years experience and peer-reviewed publications, participated in three rounds of a Delphi study to gain content consensus. After experts in each setting identified key terms in the first round, experts reviewed the consolidated list twice to generate the final competency list. Next, all certified recreational therapists were invited to rate the extent that each competency item is being used in RT practice; the extent of their knowledge/skill for each item; and the degree of interest in gaining more knowledge/skill. A total of 1377 recreational therapists participated in the study (88% females, 11% male, 1% non-disclosed) with participants ranging in age from 20-60+ years of age. The study’s findings related to specific diagnostic populations served; interventions/modalities, techniques, standardized assessment tools, and theories utilized; education, training, and counseling topics in treatment; and on-the-horizon treatment and issues. This comprehensive two-part multi-year study is the first to explore current RT practice at a micro-competency level. In the effort to improve practice, these specific competencies necessary for RT practice with older adults are significant to both educators and practitioners in future curriculum and professional development efforts.

